# What motivated men to start PrEP? A cross-section of men starting PrEP in Buffalo city municipality, South Africa

**DOI:** 10.1186/s12889-023-15306-6

**Published:** 2023-03-02

**Authors:** Philip John Smith, Joseph Daniels, Linda-Gail Bekker, Andrew Medina-Marino

**Affiliations:** 1 The Desmond Tutu HIV Centre, University of Cape Town, Observatory, Cape Town, South Africa; 2 Edson College of Nursing and Health Innovation, Arizona State University, Phoenix, AZ USA; 3 Perelman School of Medicine, University of Pennsylvania, Philadelphia, PA USA; 4 Research Unit, Foundation for Professional Development, Eastern Cape Province, 10 Rochester Rd, Vincent, East, London, Buffalo City Metro, South Africa

**Keywords:** HIV, Men, Pre-exposure Prophylaxis, South Africa

## Abstract

**Background:**

Compared to women, South African men are less likely to know their HIV status (78% vs. 89%), have suppressed viral loads (82% vs. 90%), or access HIV prevention services. To achieve epidemic control where heterosexual sexual behavior drives transmission, interventions to improve the uptake of HIV testing services (HTS) and prevention services must also target cis-gendered, heterosexual men. There is limited understanding of these men’s needs and wants with regards to accessing pre-exposure prophylaxis (PrEP).

**Methods:**

Adult men (≥ 18 years) from a peri-urban community in Buffalo City Municipality were offered community-based HTS. Those who received a negative HIV test result were offered community-based, same-day oral PrEP initiation. Men initiating PrEP were invited to participate in a study exploring men’s HIV prevention needs and reasons for initiating PrEP. An in-depth interview guide, developed using the Network-Individual-Resources model (NIRM), explored men’s perceived HIV acquisition risk, prevention needs, and preferences for PrEP initiation. Interviews were conducted by a trained interviewer in isiXhosa or English, audio-recorded and transcribed. Thematic analysis was used, guided by the NIRM to generate findings.

**Results:**

Twenty-two men (age range 18–57 years) initiated PrEP and consented to study participation. Men reported elevated HIV acquisition risk associated with alcohol use and condom-less sex with multiple partners as facilitators driving PrEP initiation. They anticipated social support from family members, their main sexual partner and close friends for their PrEP use, and discussed other men as important sources of support for PrEP initiation. Nearly all men expressed positive views of people using PrEP. Participants believed HIV testing would be a barrier for men interested in accessing PrEP. Men recommended that access to PrEP be convenient, rapid, and community-based (i.e., not clinic-based).

**Discussion:**

Self-perceived risk for HIV acquisition was a major facilitator for men’s PrEP initiation. Although men expressed positive perceptions of PrEP users, they noted that HIV testing may be a barrier to PrEP initiation. Finally, men recommended convenient access points to facilitate PrEP initiation and sustained use. Gender-responsive interventions tailored to men’s needs, wants, and voices will facilitate their uptake of HIV prevention services, and help to end the HIV epidemic.

## Introduction

With 200,000 new HIV infections annually and the world’s largest population of people living with HIV (20.4% prevalence among those aged 15–49 years), South Africa (SA) is the global epicenter of the HIV pandemic [1]. The last National HIV prevalence for South Africans aged 15–49 years was estimated at 15% and 26% among men and women respectively [2]. South African men have the highest risk of premature mortality worldwide [3], with HIV being the second leading cause of mortality after tuberculosis for those under 35 years [4]. Although women bear the brunt of HIV infections, South African men have poorer rates of HIV testing, treatment initiation, viral suppression, and survival on treatment compared to women [5–8]. While engaging men in the HIV testing, care, and treatment cascade is essential to ensuring optimal health outcomes, implementing effective HIV prevention strategies for men is crucial to curbing new HIV infections among both women and men [9].

Oral pre-exposure prophylaxis (PrEP) with emtricitabine (FTC)/tenofovir (TDF) has demonstrated significant efficacy in clinical trials [10–15]. These trials were conducted among men who have sex with men (MSM) [12], African women [10, 11], heterosexual men and women [13, 15], and injection drug users [14]. PrEP demonstration projects and government programs have prioritized key populations, including sex workers, MSM, and young women at high risk of HIV acquisition, however, there has been limited focus on cis-gender, heterosexual men’s access to PrEP outside that of studies focused on sero-discordant relationships [16–19]. In this study we will use the term “men” to denote heterosexual males. South Africa’s National Strategic Plan for HIV, TB, and STIs 2017–2022 includes the explicit aim of reducing new HIV infections through a national rollout out PrEP for all individuals, including heterosexual men [20, 21]. The guidelines recommend daily dosing for heterosexual men at high risk of HIV acquisition. However, given the lack of baseline research to understand men’s preferences for PrEP promotion and access, their uptake of PrEP services, and HIV prevention services in general, will continue to be limited [19, 22, 23].

Masculinity norms and social expectations impact men’s health behaviors, and have been linked to men’s late clinic presentation when ill and limited use of preventative health services, including HIV prevention and PrEP initiation [24–27]. Structural barriers that further impede men’s access to health and HIV services include limited resources (unemployment and poverty), the lack of male-friendly healthcare (unfriendly staff; the perception of clinics being female/ maternal-child spaces; female dominant healthcare staff), and inconvenient clinic operating hours [22, 28, 29]. Additionally, alcohol use and PrEP stigma may influence men’s PrEP persistence. In a PrEP implementation study in Cape Town, South Africa, young women noted that PrEP was desirable for HIV prevention especially in the context of alcohol use when condoms were not used or difficult to negotiate [30]. However, since alcohol use has been found to be both a facilitator and a barrier to PrEP initiation and discontinuation [31], we need to understand how alcohol impacts PrEP use in men. Lastly, the role of PrEP stigma has been explored in other populations, with findings suggesting PrEP stigma was associated with lower initiation in pregnant women [32, 33]. When provided with flexible, community-based options, men’s uptake of health services approaches that of women [22, 34, 35]. Furthermore, growing evidence indicates that men are interested in accessing sexual and reproductive health services (SRHS), including PrEP, that are delivered in community settings and cognizant of their work and life priorities [36–40]. These studies have emphasized differentiation of SRHS for men in limited resources settings in order to increase uptake of HIV services. Lastly, leveraging aspects of masculine identity, such fatherhood, may further motivate and increase men’s engagement with HIV prevention services [25].

Given South Africa’s recent policy change to expand access to PrEP for non-MSM men [18], we implemented a study that provided community-based PrEP services to men, and investigated men’s barriers and facilitators to PrEP initiation.

## Methods

### Study setting

**Fig. 1:**
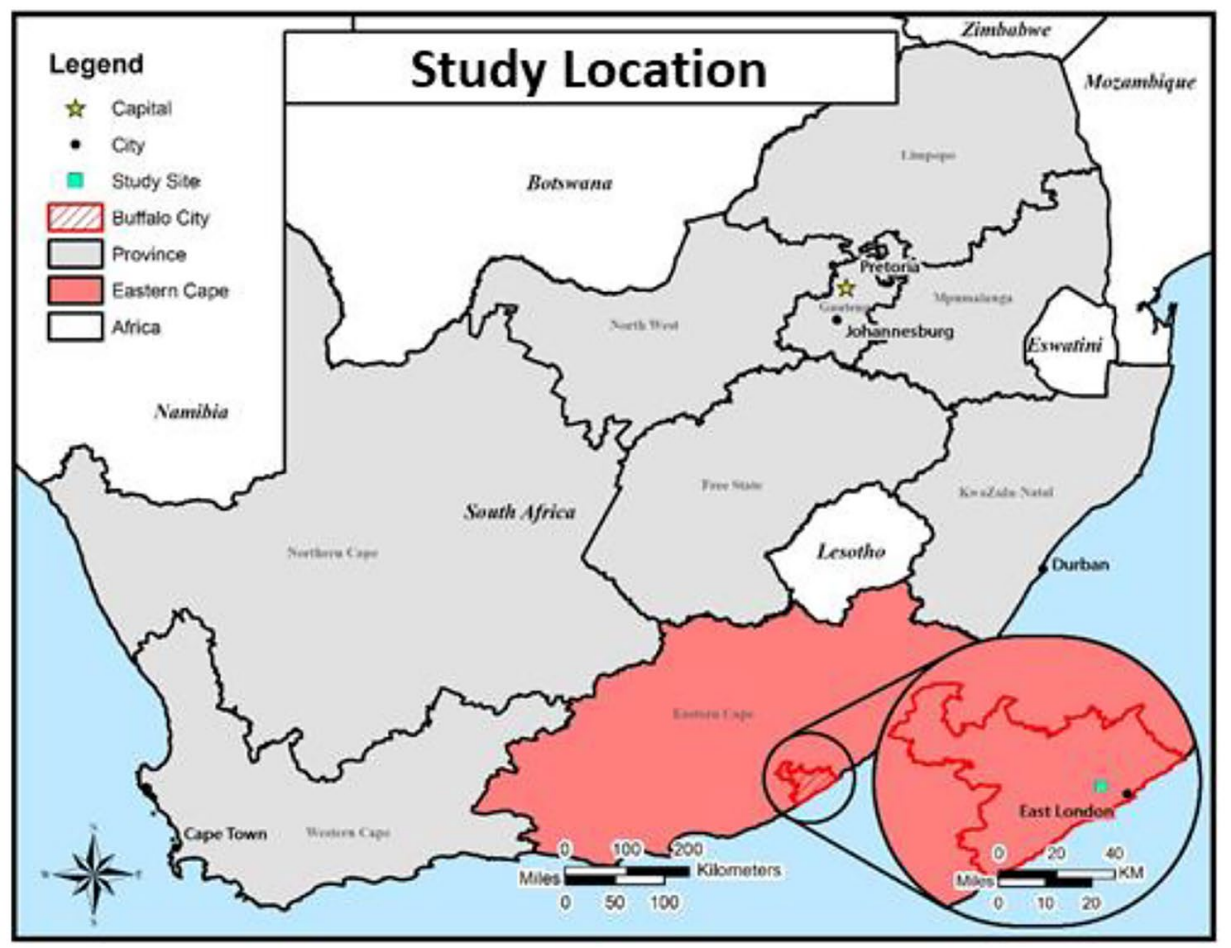
Provincial map of South Africa showing location of study community within Buffalo City Metro Health District, Eastern Cape Province

This study was nested within the Community PrEP Study (CPS) [41, 42]. CPS aimed to leverage community-based platforms to increase access and adherence to PrEP among young women in two high HIV burden communities in the Eastern Cape Province, South Africa (Fig. 1); Eastern Cape is a research naïve province with an estimated 15.3% HIV prevalence among the general population [2]. Men were recruited from the peri-urban community in which the CPS was implemented. This community is 10 km outside East London Central Business District, Buffalo City Metro Health District (BCM-HD).

### Staff training

Study staff (i.e., HIV testing service (HTS) counsellors, a fieldworker, and a study nurse) received training in the study protocol, human subjects’ protection, HIV counselling and testing protocols, good clinical practice and qualitative research methods. The fieldworker was experienced in conducting semi-structured interviews. Additionally, the fieldworker attended training on using the interview guides before study initiation.

### Participant recruitment and measures

**Fig. 2:**
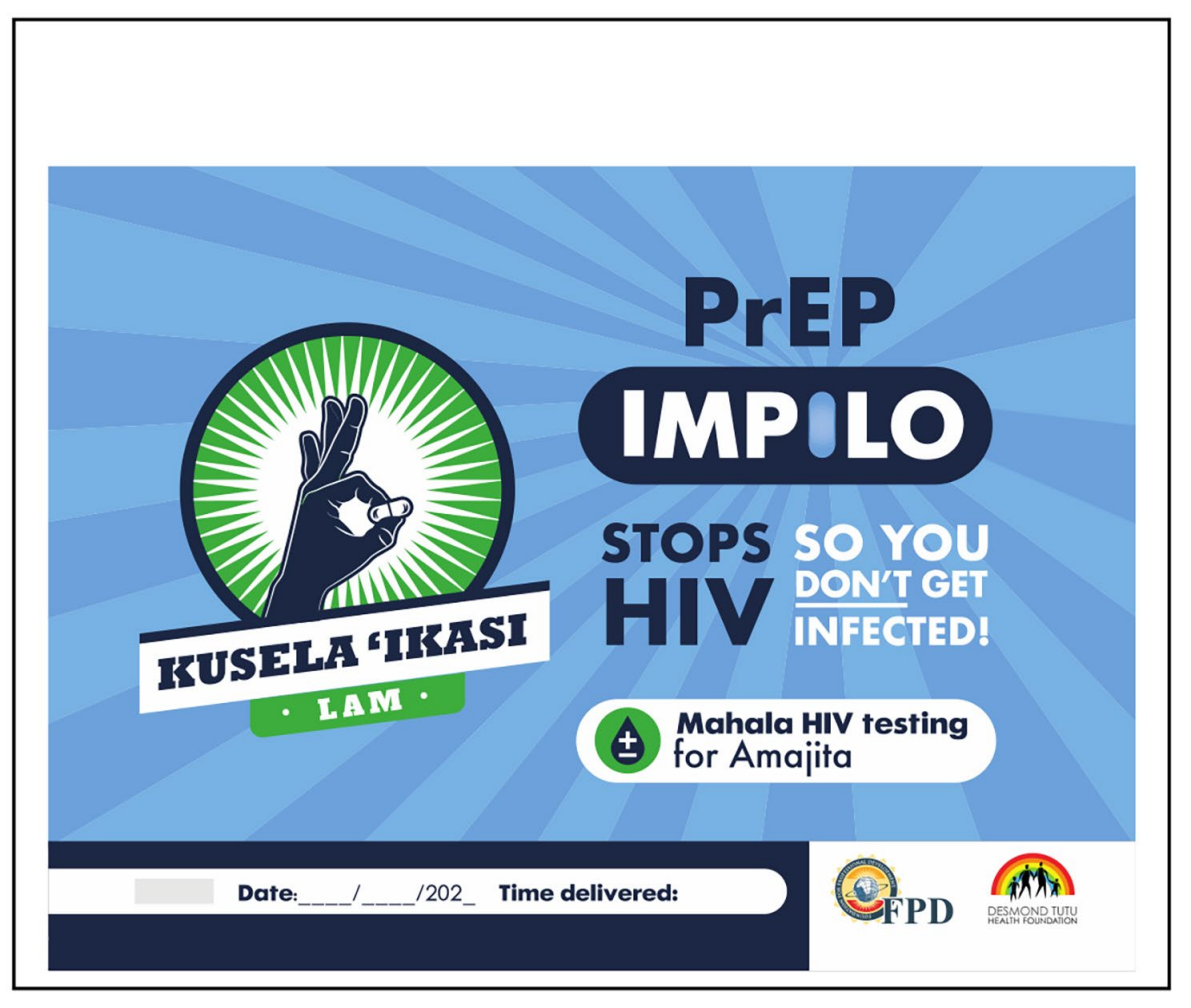
Invitation flyer for the Male Community PrEP Study

The HIV testing recruiters handed out invitation cards (Fig. 2) [43] to men walking within 500 m of the mobile gazebo HIV testing site. Recruitment took place between 09:30 and 15:30 on weekdays. Upon presentation, HTS counselors offered all individuals (i.e., men and women) an HIV test per South African National HIV testing standards [44]. Study eligibility was defined by the following inclusion criteria: self-identified as male, aged ≥ 18, confirmed HIV negative test result at screening, interest in taking PrEP, provision of written informed consent. Men who received an HIV positive test result were immediately referred for clinical care and ART services at the local clinic per South African National Guidelines. Men aged ≥ 18 years, with an HIV negative test result were invited to learn about PrEP. Those expressing interest in PrEP were referred to the study-established community-based PrEP services site which was co-located in the same community.

A trained fieldworker read aloud an informed consent form to all eligible and interested participants. After obtaining written informed consent, the fieldworker allocated a study ID number to the participant and then administered a survey which included questions related to the participant’s socio-demographics, the PrEP stigma scale [45], and the Alcohol Use Disorders Identification Test (AUDIT-C) [46]. The PrEP stigma scale had 13 items, requiring participants to rate how much they agree with statements along a five-point Likert-type scale from “strongly disagree” to “strongly agree”. The first item on the scale is, “I would feel ashamed to take PrEP pills in front of others”. The fieldworker then interviewed participants using a semi-structured, in-depth interview (IDI) guide; interviews were conducted in a participant’s preferred language (English or isiXhosa). The IDI guide was developed in English and was translated into Xhosa by an isiXhosa speaking researcher trained in qualitative research methods. The principal investigator and the researcher discussed the translation and reviewed the guide with the fieldworker for comprehensibility by research participants. IDIs explored men’s: (1) decision making regarding PrEP initiation; (2) perceptions of other people taking PrEP, (3) perceptions of other men’s barriers and facilitators regarding PrEP uptake/initiation, and (4) preferences for uptake and access to PrEP refills. Upon completion of the IDI, a nurse collected blood samples for creatinine (not reported) as a marker of kidney functioning and STI diagnostic testing (chlamydia trachomatis, gonorrhea, syphilis). STI results were used as a clinical marker of sexual risk for HIV acquisition. Participants who were diagnosed with an STI were informed and referred for treatment. The nurse conducted counselling around PrEP initiation and adherence and provided participants with one month’s supply of PrEP and R100 (~$6.60) for their time and transport costs.

### Theoretical framework

The network-individual-resource model (NIRM) informed the development of the IDI guide and result contextualization. The NIRM takes an ecological approach to understanding behavior [47]. The model was developed to inform HIV prevention interventions, and has been adapted for use in other contexts in South Africa [48, 49, 50]. The NIRM articulates the role that mental (e.g., psychosocial, cognitive, social support, social cues, perceived or internalized stigma, perceived or actual norms, self-perceived masculinity, mental health, knowledge/education) and tangible (e.g., income, physical health, access to health services, food security, money) resources play in health behaviors, decisions, and outcomes, and was used to understand what resources men associate with PrEP initiation. Accordingly, PrEP initiation will depend on the ability of men to (1) locate and leverage tangible resources (i.e., work, access to health services) and (2) access mental resources (i.e., support from family, male friends and partners) [47]. Moreover, the NIRM was used to understand how men thought about their tangible and mental resources, and how these resources influenced their perceived HIV risk, prevention discussions with partners, and PrEP use behaviors.

### Data analysis

Audio-recorded interviews were transcribed into English. A bilingual researcher trained in qualitative research methods translated the interview audio. The translations were discussed with the PI where phraseology was clarified where needed. Using thematic analysis [51, 52], the first five transcripts were read by two researchers and open coded to identify mental and tangible resource influential in PrEP initiation and adherence [47]. After review by the research team, codes were assembled into a codebook that was applied to all transcripts. Once coding was completed, a frequency analysis was conducted to generate a range of resources from most to less frequently discussed, which were subsequently organized into matrices to understand positive and negative influences on PrEP initiation and adherence [53]. This quantification was conducted to determine most frequently cited resources, and to create a useful matrix for organizing the analysis and interpretation. Memo writing and causal diagrams were developed to refine preliminary themes that were presented to the research team for discussion, informing additional analysis to generate the final themes [24, 54].

### Ethics and participant representation

In accordance with the Declaration of Helsinki (1964), all research was conducted adhering to ethical guidelines and the study was approved by the Human Research Ethics Committee at the University of Cape Town (HREC Ref 173/2021). Approval to conduct research in the selected sites was provided by the Eastern Cape Provincial Department of Health, and supported with local approval by a Community Advisory Board. Written informed consent was obtained from all participants before enrolling in the study. Participant quotes are represented by age and a participant ID number to ensure confidentiality.

## Results

We enrolled 22 participants’ (Median Age: 24 years; IQR: 20–33). Participant characteristics are reported in Table 1. Most participants reported neutral levels of PrEP stigma [45], but moderate to severe levels (86%) of hazardous alcohol consumption.

**Table 1:** Participant characteristics

	n	%
**Median age years**	24	IQR 20–33
18–19	4	18%
20–24	7	32%
25–29	3	14%
30–34	2	9%
35–39	4	18%
≥ 40	2	9%
PrEP Stigma*	3.1	
**AUDIT-C**		
Severe Risk : 8–12 points	5	23%
High Risk: 6–7 points	6	27%
Moderate Risk : 4–5 points	8	36%
Low Risk: 0–3 points	3	14%
**STI**		
Chlamydia trachomatis	4	18%
Gonorrhoea	0	0%
Syphilis	0	0%

*Scale score ranges from 1–5, where higher values indicate higher stigma

While there were limited codes specific to individual participants, these codes were not sufficient to generate new themes. Additionally, while participants tended to frame their responses personally, some responses presented a collective perspective denoted by “we” and “men”. Interviews generated four key findings: (1) men’s self-perceived HIV risk motivates their interest in PrEP; (2) HIV testing hesitancy and clinic access are barriers to PrEP initiation; (3) men are hearing about PrEP from women and people in the gay, men-who-have-sex-with-men (MSM) and transgender communities; and (4) clinic proximity to working and living locations and friendly staff will promote PrEP uptake.

### Men’s self-perceived HIV risk motivates their interest in PrEP

Motivating their interest in PrEP, men described risk behaviors including: alcohol consumption; sexual behaviors; multiple sexual partners (MSP); and limited discussion of HIV prevention practices with sexual partners.

Participants frequently linked alcohol consumption to their desire for engaging in sex. Men further described prioritizing sex in the moment over discussing prevention, especially if condoms were not available. They knew that unprotected sex increased their risk for HIV acquisition, and even described being anxious about HIV infection after having unprotected sex.

*Participant 6, 19 years: “I would say it is very high [HIV risk], because they like alcohol, and we as men we have this tendency, we are drunk, we like women, we must have sex. So, others take that advantage that they are drunk now, so we must have sex. So, that is the reason I say the rate of HIV is very high, alcohol is the influence, because everything they do they are not in their right minds because of alcohol, so they do whatever they want to do.”*

*Participant 2, 25 years: “When we are drunk, we are men that just do things without consideration. Too much, because the minute you have alcohol you think too much and end up saying that my partner is not here and I can do this, no one is going to see me, do you understand? So that is when we end up taking risks.”*

*Participant 18, 41 years: “… when you meet with someone and the condom is not there, then you just decide to just continue. After the act, you just realize that you did not use condom.”*

*Participant 17, 20 years: [HIV prevalence] is very high. It is, my brother, because we talk about everything and we don’t use condoms.”*

Participants reported that MSP was common, and correctly associated MSP with increasing one’s risk for HIV acquisition. Participants described how the practice of MSPs resulted in confusion and a lack of trust in their own relationships. One participant even wondered aloud whether their partner had other partners. Being unsure about trust in relationships caused the participant to think about how to ensure his own safety and the safety of his partner.

*Participant 4, 27 years: “Bro the small number of people I know introduce me to people’s partners but when I come back I find that, that person is no longer with that person and that they are now with someone else. That leaves me sort of confused, otherwise I think the risk of HIV here is high.”*

*Participant 4: “Earlier I spoke to [the nurse] and spoke about trust. You can trust someone but you don’t know them fully. For instance, I was in [a neighbouring city] and my partner is here and I don’t remember what she [the nurse] asked me, but my answer was I don’t know. I think she [the nurse] asked if she [partner] doesn’t have another person and my answer was I don’t know. So, it would really suck to find out that she has another person through an HIV test, finding out that she has HIV. What comes to my mind is her safety and mine because I don’t 100% know her and the things she is doing when I am not around.”*

### Clinic access barriers and hesitancy to test for HIV

Men were asked to describe their use of clinic-based health services and previous awareness of PrEP. Men stated that they avoid clinics for various reasons, including unfriendly staff, long waits, and a lack of privacy; some men even described leaving a clinic without being helped. One participant described the clinic as “not safe” because the clinic staff treated patients poorly and they were not respectful of patients.

*Participant 16, 19 years: “Clinics don’t treat people well, as a result men don’t want to go to clinic because it is not safe. The main problem is spending the whole day at the clinic and you end up leaving without getting any help.”*

*Participant 22, 39 years: “It’s very nice coming here [the research site] because you won’t have to face the community like in the clinic meeting people with different problems and here it’s only based on HIV so that is why I like it and you get less judges by the community.”*

One significant barrier to PrEP was the need to be tested for HIV. One participant stated that men worry about testing positive for HIV after having unprotected sex, and that this fear factored into avoiding HIV testing. Another participant noted that women routinely visited clinics and were offered HIV testing and HIV prevention, adding that men do not routinely visit clinics, and HIV prevention was understood to be for women.

*Participant 16, 19 years: “I can’t say those people [in the community] protect themselves and men don’t go to the clinics, it’s mostly women, and we don’t know the statistics.”*

*Participant 1, 35 years: “Men are afraid to do testing and things that are associated with HIV (illness). It is not easy, but the way you have approached us is one (delivering invitations), I don’t know any other way you could have done. You have to be ready before you decide to go and test, so it is difficult in that way.”*

*Participant 11, 19 years: “We do things then get scared of the outcomes, for example having sex without a condom and then fearing taking PrEP because you will get tested for HIV, and think what if it is positive. We don’t want to know.”*

### Men are hearing about PrEP from women and people in the gay, MSM and transgender community

Most men were not completely ignorant about PrEP, stating that they had heard about PrEP from a partner or a family member, or overheard people talking about it. Men specifically noted that their knowledge of PrEP was obtained through women or the gay-bisexual-MSM-transgender community. However, men thought PrEP was only for ‘certain individuals’ (i.e., women and men who have sex with men), and not them.

*Participant 4, 27 years: “Now that you mention, my girlfriend, she once told me about it but she told me about its side effects, how she felt when she took it. Uhm she was not alright, she felt like she was going to vomit and dizzy.”*

*Participant 14, 34 years: “I overheard people talking about it and in most cases it’s gays. What were they saying, uhm, in a way people that are sexually active, they are, or should I say we are, using a condom is, I don’t want to say is the last thing on our minds, it’s not the first thing on our minds… so they say a lot of things to each other and I hear them talking about that, and maybe they are talking and saying PrEP is available and so on, things like that.”*

*Participant 4: “Transgenders have female minds and say a lot to each other. So, I heard them talking, saying that we will not die from AIDS while PrEP is there. So, that is how I heard about it, but for someone telling me about PrEP clearly, it is [the recruiter].”*

Some men heard messaging that they shouldn’t take PrEP. One participant mentioned that people in his community have discouraged the use of medication, and when asked about barriers to PrEP initiation, one participant stated, “there are people that are discouraging the use of other things like the [COVID19] vaccine.” Consequently, men who are exposed to skepticism may experience this as a barrier to PrEP initiation if skepticism is normative.

Participants expressed a positive perception towards those who choose to use PrEP. Specifically, men understood that people took PrEP to minimize their HIV risk, and universally described those individuals as being responsible. Such perceptions may be a facilitating factor for men’s own PrEP initiation and use. However, they also acknowledged the existence of PrEP-related stigma, which may be related to the perception that PrEP is a treatment for HIV.

*Participant 10, 32 years: “I think it is being responsible, I don’t look at them as wanting to be loose but being responsible. At the end of the day, even if they don’t take PrEP, if they decide to have multiple partners, they will do that, so rather they be safe.”*

*Participant 6, 19 years: “That’s a great thing that they are doing. They are protecting themselves and their partners.”*

*Participant 11, 19 years: “They don’t want to be HIV infected, and they are taking responsibility for their lives. They want to be safe.”*

### PrEP access close to their living and working locations that is administered by relatable individuals will ensure initiation

Quick access to PrEP services was a significant factor influencing PrEP uptake. Men discussed how the convenience of community-based HIV testing and PrEP services, both in terms of duration of visit and location, influenced their uptake of these services. Men frequently noted the importance of a PrEP service location being close to where they lived, or accessing PrEP through delivery or pharmacy pickup due their work hours. The study’s community-based location, compared to clinic-based services, was also seen as a benefit because it did not entail having to see people or explain the reason for one’s visit to a clinic.

*Participant 1, 35 years: “I don’t have to go to town for other things because they are here. And also this PrEP thing, I don’t need to go and look for it far.”*

*Participant 22, 39 years: [quotation used previously] “It’s very nice coming here [the research site]…*

*Participant 1, 35 years: “I have spoken with one of these men and he said, he would prefer it to be delivered or get prescription so that he can get it at the chemist because of work.”*

Participants did not express strong preferences for male or female clinic staff, but mentioned that it was important for staff to be relatable and professional.

*Interviewer: “Alright, when accessing PrEP, would you like a man or a lady on your pick- ups?*

*Participant 9, 22 years: I would say both [male and female staff] if they are professional, I would [go] to either but only if they are professional.”*

*Participant 17, 20 years: “From both [male and female]. There is no difference, you came for the treatment.”*

## Discussion

Daily PrEP has been recommended by the South African Department of Health for populations at risk of HIV acquisition, including heterosexual men [20, 21]. However, few studies have reported on cis-gender, non-MSM men’s preferences for access to PrEP and PrEP services or the mental and tangible resources this population would use to initiate PrEP and sustain use. Our study reports that men did not think that PrEP was for or available to them, as they had heard about PrEP from women in their lives (partners or family), or overheard gay, MSM or transgender community members discussing PrEP. Though participants were unaware that PrEP was for them, risk perception was a key mental resource that motivated of their interest in and initiation of PrEP. However, men reported that HIV testing would likely be a major barrier to PrEP uptake for other men. Finally, convenience was a tangible resource for participants, given that men recommended that PrEP services be close to home or work commute, have accessible timing, and have rapid consultations.

While men were generally aware of PrEP through partners, family members, or by overhearing conversations in their community, it was not commonly understood to be an option for them. Even so, men expressed little stigma towards other PrEP users. In fact, their perception of those taking PrEP was positive, stating that PrEP users were being responsible and protecting their own health and that of their partner(s). Such low stigma and positive attributes likely served as mental resources for their own decisions to use PrEP. Since men noted that they had heard about PrEP from their familial or social networks, community-based PrEP champions may serve as important role models for initiating and continuing PrEP use [55].

Participants became aware that PrEP was initially targeted to MSM, adolescent girls and young women. Now that PrEP was being targeted to them, they felt it critical to ensure that access to PrEP services also spoke to their needs. Specifically, men discussed how and why community-based services and locations appealed to their needs, and how clinic-based services were sub-optimal due to poor staff attitudes, perceived female spaces and long wait times. However, even with optimized access to PrEP services, HIV testing was seen as a major deterrent and barrier to accessing PrEP, as men feared receiving an HIV positive test result. Since the interview was also designed to explore barriers that may limit other men from accessing PrEP. Participants noted that HIV testing may be a barrier for other men to initiate PrEP. In the interviews, exploring the barriers was designed to explore what other men may perceive to limit or prevent PrEP access. To reduce this barrier, HIV testing messaging should emphasize the benefits of testing, and the services available to men when they know their HIV status [43, 56]. Tailored and convenient services and tailored messaging were tangible resources that would support PrEP uptake. Moreover, messaging that addresses fears around HIV testing would mentally support men by relieving anxiety associated with HIV testing.

Given men’s mental/ knowledge resource of the association between alcohol use, MSP and HIV acquisition, incorporating messages about how PrEP may protect them even when they engage in these behaviors may improve their mental model of how to protect themselves against HIV and promote PrEP initiation. Furthermore, acknowledging risk associated with alcohol use may be an important mental resource for communicating the benefits of PrEP. Consequently, disseminating PrEP promotion messaging within bars, taverns and shebeens may allow for targeting those men with these specific risks [57]. Non-venue-based community information campaigns can incorporate the specific risk factors identified by men as reasons for PrEP use, as well as the benefits of PrEP articulated by men. Finally, given that men initially heard about PrEP via their social networks or overheard conversations in their communities, leveraging social and community networks (tangible and mental resources) and gorilla marketing-style campaigns should be considered [58].

### Limitations

While this study aimed to understand men’s preferences for PrEP initiation, only men who elected to be tested for HIV and initiated PrEP were included. Given participant’s comments about HIV testing being a barrier for other men, the lack of voice of those men limits our understanding of how best to engage men in PrEP services. Some of the descriptions provided by participants reported on their perception of other mens’ barriers to testing and PrEP uptake. While illuminating, these descriptions should be considered secondary findings with lower validity. Towards this, future studies should seek to identify men interested in PrEP, but for whom HIV testing is a barrier. Moreover, our findings are based on interviews conducted at PrEP initiation. However, motivations for sustaining PrEP use may change over time. This limitation may be addressed in future studies by serial interviews comparing the initial assumptions about motivations to use PrEP versus what they actually found influenced their sustained PrEP use. It is important to note that while men’s awareness of their alcohol related risk motivated uptake in this study, excessive alcohol use is ordinarily associated with reduced health behaviour. Even though it may be beneficial to consider the recommendation to market PrEP at taverns and shebeens, our interview guide did not investigate the potential effect of alcohol use on sustained PrEP use. The study did not track participation rate among those who were approached, or reasons for declining to participate. Recording declination and reasons may be informative in future studies. Following on, the study was conducted during work hours, which may have prevented those who work during these times from participating. Finally, given that our sample size was small and recruitment was not random, caution should be employed when transferring findings beyond the study. The study targeted a high HIV disease burden community in the Eastern Cape. While participants from this location may have shared similarities with men from other communities, including more locations in future investigations may provide better transferability of the findings. Although the sample size may have been a limitation, there were no additional themes generated in the final interviews. Specifically, there were codes that were unique to individual interviews, but these were not sufficient to create new themes. This may indicate that thematic saturation was reached.

## Conclusion

Although men in this study exhibited interest in PrEP uptake, there are significant barriers to their healthcare access where PrEP is currently available. Some common barriers included the need to test for HIV and reticence towards visiting a conventional healthcare facility. Furthermore, while alcohol use was not noted as a barrier to PrEP uptake, excessive alcohol use is associated with reduced health-seeking behavior [17, 57]. Future studies need to investigate the utility of counselling men about common barriers and pathways to accessing healthcare. Additionally, interventions would greatly benefit men by including education around mental models for overcoming barriers and attaining desirable outcomes.

## Data Availability

The data generated and analysed during the current study are available from the corresponding author on reasonable request.
